# Non-contiguous finished genome sequence and description of *Gorillibacterium massiliense* gen. nov, sp. nov., a new member of the family *Paenibacillaceae*

**DOI:** 10.4056/sigs.5199182

**Published:** 2014-03-15

**Authors:** Mamadou Bhoye Keita, Roshan Padhmanabhan, Aurélia Caputo, Catherine Robert, Eric Delaporte, Didier Raoult, Pierre-Edouard Fournier, Fadi Bittar

**Affiliations:** 1 URMITE, Aix-Marseille Université, Faculté de médecine, Marseille, France; 2 IRD, University Montpellier 1, Montpellier, France; 3 King Fahad Medical Research Center, King Abdul Aziz University, Jeddah, Saudi Arabia

**Keywords:** *Gorillibacterium massiliense*, genome, culturomics, taxono-genomics

## Abstract

Strain G5^T^ gen. nov., sp. nov. is the type strain of *Gorillibacterium massiliense,* a newly proposed genus within the family *Paenibacillaceae*. This strain, whose genome is described here, was isolated in France from a stool sample of a wild *Gorilla gorilla* subsp. *gorilla* from Cameroon. *G. massiliense* is a facultatively anaerobic, Gram negative rod. Here we describe the features of this bacterium, together with the complete genome sequence and annotation. The 5,546,433 bp long genome (1 chromosome but no plasmid) contains 5,145 protein-coding and 76 RNA genes, including 69 tRNA genes.

## Introduction

Strain G5^T^ (= CSUR P290 = DSM 27179) is the type strain of *Gorillibacterium massiliense* gen. nov., sp. nov. This bacterium which is proposed to belong to the family *Paenibacillaceae*, is a Gram-negative, flagellated, facultative anaerobic, indole-negative bacillus that was isolated from a fecal sample of a wild western lowland gorilla from Cameroon, through a culturomics study of the bacterial diversity of the feces of wild gorillas. This technique was used successfully to explore the human gut microbiota allowing the isolation of many new species and genera [1-3].

The newly proposed strategy of applying high throughput genome sequencing, MALDI-TOF spectral analysis of cellular proteins, coupled with more traditional methods of phenotypic characterization has been demonstrated as a useful approach for the description of new bacterial taxa [4-15]. A principle advantage is that this method circumvents the vagaries of methods that rely mainly on DNA-DNA hybridization to delineate species. Here, we applied this polyphasic approach to describe *G. massiliense* gen. nov., sp. nov. strain G5^T^.

The family *Paenibacilliaceae* [16] belongs to the phylum *Firmicutes* and includes the 9 following genera [17]: *Paenibacillus* [18,19], *Ammoniphilus* [20], *Aneurinibacillus* [21], *Brevibacillus* [21], *Thermobacillus* [22], *Fontibacillus* [23], *Cohnella* [24], *Saccharibacillus* [25] and *Oxalophagus* [26]. Members belonging to this family were isolated mainly from soil, roots, blood, feces and other sources [16]. To the best of our knowledge, this is the first report of the isolation of a novel genus from the fecal flora of a gorilla.

Here we present a summary classification and a set of features for *G. massiliense* gen. nov., sp. nov. strain G5^T^ (= CSUR P290 = DSM 27179) together with the description of the complete genomic sequencing and its annotation. These characteristics support the circumscription of a novel genus, *Gorillibacterium* gen. nov. within the family *Paenibacillaceae*, with *Gorillibacterium massiliense* gen. nov., sp. nov. as the type species.

## Classification and features

**Figure 1:**
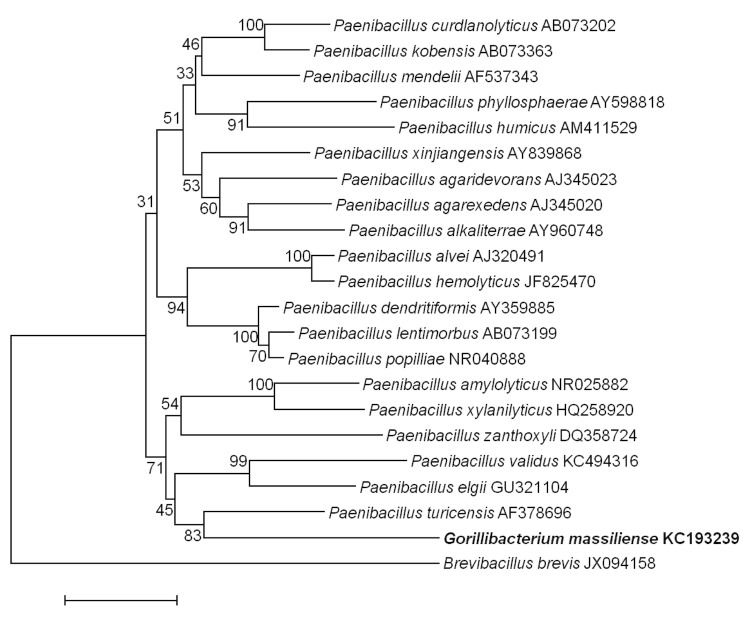
Phylogenetic tree highlighting the position of *Gorillibacterium massiliense* strain G5^T^ relative to other type strains within the *Paenibacillaceae* family. GenBank accession numbers are indicated in parentheses. Sequences were aligned using CLUSTAL X (V2), and phylogenetic inferences obtained using the maximum-likelihood method within the MEGA 5 software [36]. Numbers at the nodes are percentages of bootstrap values obtained by repeating the analysis 1,000 times to generate a majority consensus tree. *Brevibacillus brevis* was used as out-group. The scale bar represents a 2% nucleotide sequence divergence.

In July 2011, a fecal sample was collected from a wild *Gorilla gorilla* subsp.* gorilla* near Minton, a village in the south-central part of the DJA FAUNAL Park (Cameroon). The collection of the stool sample was approved by the Ministry of Scientific Research and Innovation of Cameroon. No experiments were conducted on this gorilla. The fecal specimen was preserved at -80°C after collection and sent to Marseille. Strain G5^T^ (Table 1) was isolated in August 2012 by aerobic cultivation at 37°C on sterilized soil medium (12 g of soil (Latitude: N 43° 17' 20.151''; Longitude: E 5° 24' 15.3822'') /agar (14g/l). This strain exhibited a 93.72% 16S rRNA nucleotide sequence similarity with *Paenibacillus turicensis*, the phylogenetically closest validly published *Paenibacillus* species (Figure 1). This value was lower than the 95.0% 16S rRNA gene sequence threshold recommended by Stackebrandt and Ebers to delineate a new genus without carrying out DNA-DNA hybridization [37].

**Table 1:** Classification and general features of *Gorillibacterium massiliense* strain G5^T^

**MIGS ID**	**Property**	**Term**	**Evidence code^a^**
	Current classification	Domain *Bacteria*	TAS [27]
		Phylum *Firmicutes*	TAS [28-30]
		Class *Bacilli*	TAS [31,32]
		Order *Bacillales*	TAS [33,34]
		Family *Paenibacillaceae*	TAS [16,32]
		Genus *Gorillibacterium*	IDA
		Species *Gorillibacterium massiliense*	IDA
		Type strain G5^T^	IDA
	Gram stain	Negative	IDA
	Cell shape	rod	IDA
	Motility	non-motile	IDA
	Sporulation	non-sporulating	IDA
	Temperature range	mesophilic	IDA
	Optimum temperature	37°C	IDA
MIGS-6.3	Salinity	no Growth in BHI medium + 5% NaCl	IDA
MIGS-22	Oxygen requirement	facultative anerobic	IDA
	Carbon source	varied (see Table 2)	IDA
	Energy source	Chemoorganoheterotrophic	IDA
MIGS-6	Habitat	gorilla gut	IDA
MIGS-15	Biotic relationship	free living	IDA
MIGS-14	Pathogenicity Biosafety level Isolation	Unknown 2 Gorilla feces	NAS NAS IDA
MIGS-4	Geographic location	Cameroon	IDA
MIGS-5	Sample collection time	July 2011	IDA
MIGS-4.1	Latitude	2.783938	IDA
MIGS-4.1	Longitude	13.030472	IDA
MIGS-4.3	Depth	surface	IDA
MIGS-4.4	Altitude	> 600 m above sea level	IDA

^a^Evidence codes - IDA: Inferred from Direct Assay; TAS: Traceable Author Statement (i.e., a direct report exists in the literature); NAS: Non-traceable Author Statement (i.e., not directly observed for the living, isolated sample, but based on a generally accepted property for the species, or anecdotal evidence). These evidence codes are from the Gene Ontology project [35]. If the evidence is IDA, then the property was directly observed for a live isolate by one of the authors or an expert mentioned in the acknowledgements.

**Figure 2:**
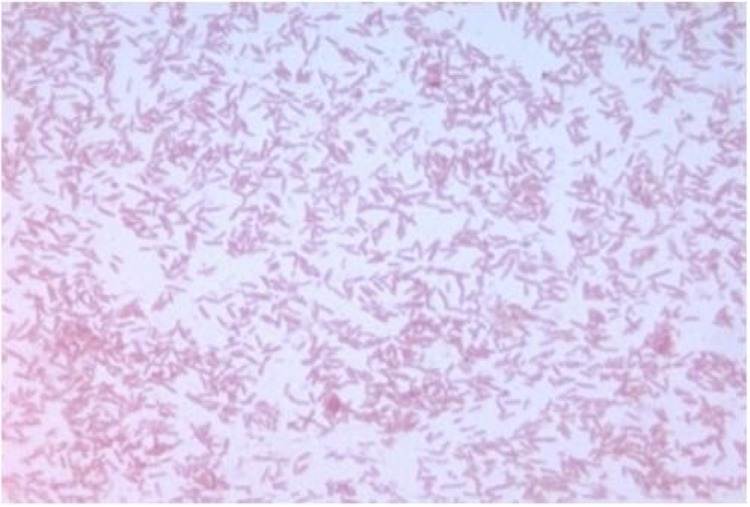
Gram staining of *G. massliensis* strain G5^T^.

**Figure 3:**
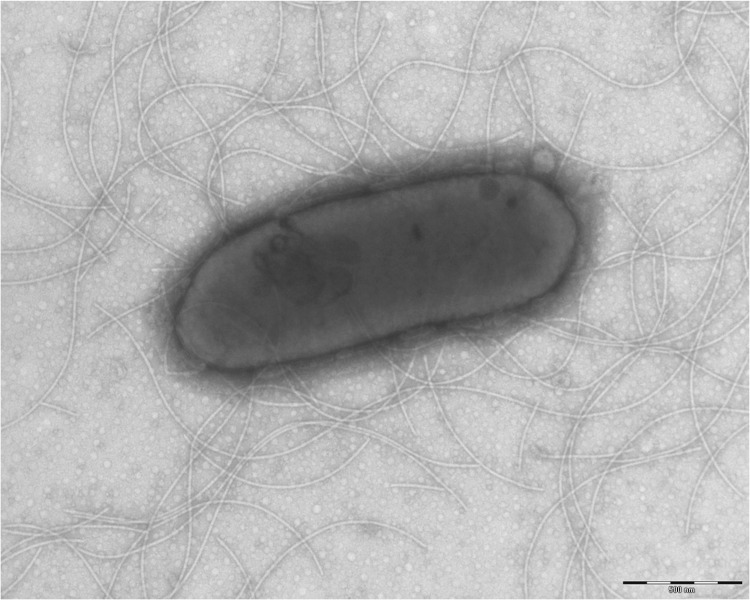
Transmission electron microscopy of *G. massiliense* strain G5^T^ using a Morgani 268D (Philips) at an operating voltage of 60kV. The scale bar represents 500 nm.

Different growth temperatures (25, 30, 37, 45°C) were tested. No growth occurred at 45°C, growth occurred between 25°and 37°C, and optimal growth was observed at 37°C. Colonies were bright grey with a diameter of 1.0 mm on 5% blood-enriched Columbia agar. Growth of the strain was tested under anaerobic and microaerophilic conditions using GENbag anaer and GENbag microaer systems, respectively (BioMérieux), and under aerobic conditions, with or without 5% CO_2_. Growth was observed under anaerobic and microaerophilic conditions, but optimal growth was obtained aerobically. Moreover, the Gram staining showed Gram-negative rod (Figure 2). A motility test produced a negative result. Cells grown on agar did not sporulate and the rods exhibited peritrichous flagella and had a mean length of 1.75 µm and a mean diameter of 0.67 µm as determined by negative staining transmission electron microscopy (Figure 3).

Strain G5^T^ exhibited catalase activity but not oxidase activity. Using the API 50CH system (BioMerieux), a positive reaction was obtained for D-xylose, D-glucose, D-fructose, D-mannose, N-acetylglucosamine, aesculin, salicin, D-cellobiose, D-maltose, D-lactose, D-melibiose, D-saccharose, D-trehalose, inulin, D-melezitose, D-raffinose, glycogen, gentiobiose, D-turanose, Methyl-α-D-glucopyranoside and hydrolysis of starch. A weak positive reaction was observed for L-arabinose. A negative reaction was observed for glycerol, ribose, D-galactose, L-rhamnose, L-sorbose, dulcitol, inositol, D-mannitol, D-sorbitol, methyl-αD-mannopyranoside, D-arabinose, amygdalin, arbitin, potassium gluconate, potassium 2-cetogluconate, potassium 5-cetogluconate, adonitol and D-tagatose. Using the API ZYM system, positive reactions were obtained only for naphthol-AS-BI-phosphohydrolase, α-galactosidase, β-galactosidase, β-glucosidase, arginine arylamidase and arginine dihydrolase. The production of α-glucosidase, β-glucuronidase, esterase lipase, leucine arylamidase, cystine arylamidase, valine arylamidase, glycine arylamidase, phenylalanine arylamidase, lipase, alkaline phosphatase, acid phosphatase, N-acetyl-β-glucosaminidase and a-chymotrypsin were negative. Urease reaction and reduction of nitrates to nitrogen were also positive. Indole production was negative. *G. massiliense* was susceptible to ticarcillin, amoxicillin, tobramycin, imipenem, vancomycin and rifampin but resistant to ceftazidime (Caz 30), colistin (CT50) and metronidazole.

When compared with representative species from the family *Paenibacillaceae* [38-42], *G. massiliense* gen. nov., sp. nov. strain G5^T^ exhibited the phenotypic differences detailed in Table 2.

**Table 2:** Differential phenotypic characteristics between *Gorillibacterium massiliense* gen. nov., sp. nov., strain G5^T^ and phylogenetically close *Paenibacillaceae* species.

**Characteristic**	1	2	3	4	5
Gram stain	-	+	var	var	+/var
Motility	-	+	+	+	+
Endospore formation	-	+	+	+	+
Isolated from	Gorilla gut	Human: valve of a cerebrospinal fluid shunt	Roots of *Perilla frutescens*	Honeybee larvae	Environment: soil
**Production of**					
Catalase	+	-	+	+	+
Oxidase	-	-	-	+	+
Nitrate reductase	+	-	+	-	+
Urease	+	-	+	na	-
Indole	-	-	+	+	-
**Utilization of:**					
Glycerol	-	-	var	-	-
D-xylose	+	+	var	-	-
D-glucose	+	+	+	+	-
D-fructose	+	+	w	na	-
D-mannose	+	+	+	-	-
Methyl- αD-mannopyranoside	-	-	na	na	-
N-acetylglucosamine	+	+	+	na	+
Aesculin	+	+	+	na	+
Salicin	+	+	-	-	-
D-cellobiose	+	+	+	-	+
D-maltose	+	+	+	na	-
D-lactose	+	+	+	na	-
D-melibiose	+	+	na	+	-
D-saccharose	+	+	na	-	-
D-trehalose	+	-	+	-	-
D-melezitose	+	-	na	-	-
D-raffinose	+	+	na	+	-
Starch	+	+	+	+	-
Glycogen	+	+	w	-	-
β-Gentiobiose	+	+	w	na	-
L-arabinose	w	+	-	-	-
Ribose	-	+	+	na	-
D-galactose	-	+	+	na	+
D-mannitol	-	-	+	-	+
Potassium gluconate	-	-	+	-	w
Amygdalin	-	+	-	-	-

Strain: 1, *Gorillibacterium massiliense* G5^T^; 2, *Paenibacillus turicensis* MOL722^T^; 3, *Paenibacillus elgii* SD17^T ^; 4, *Paenibacillus alvei* BCRC 11220^T^; 5, *Brevibacillus brevis* NBRC 15304^T^.

-: negative result, +: positive result, var: variable, na: data not available, w: weak positive result

**Figure 4:**
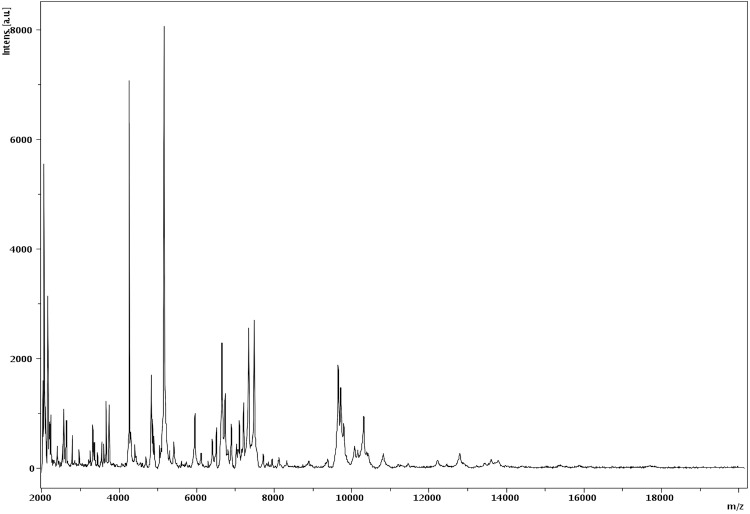
Reference mass spectrum from *G. massiliense* strain G5^T^. Spectra from 16 individual colonies were compared and a reference spectrum was generated.

**Figure 5:**
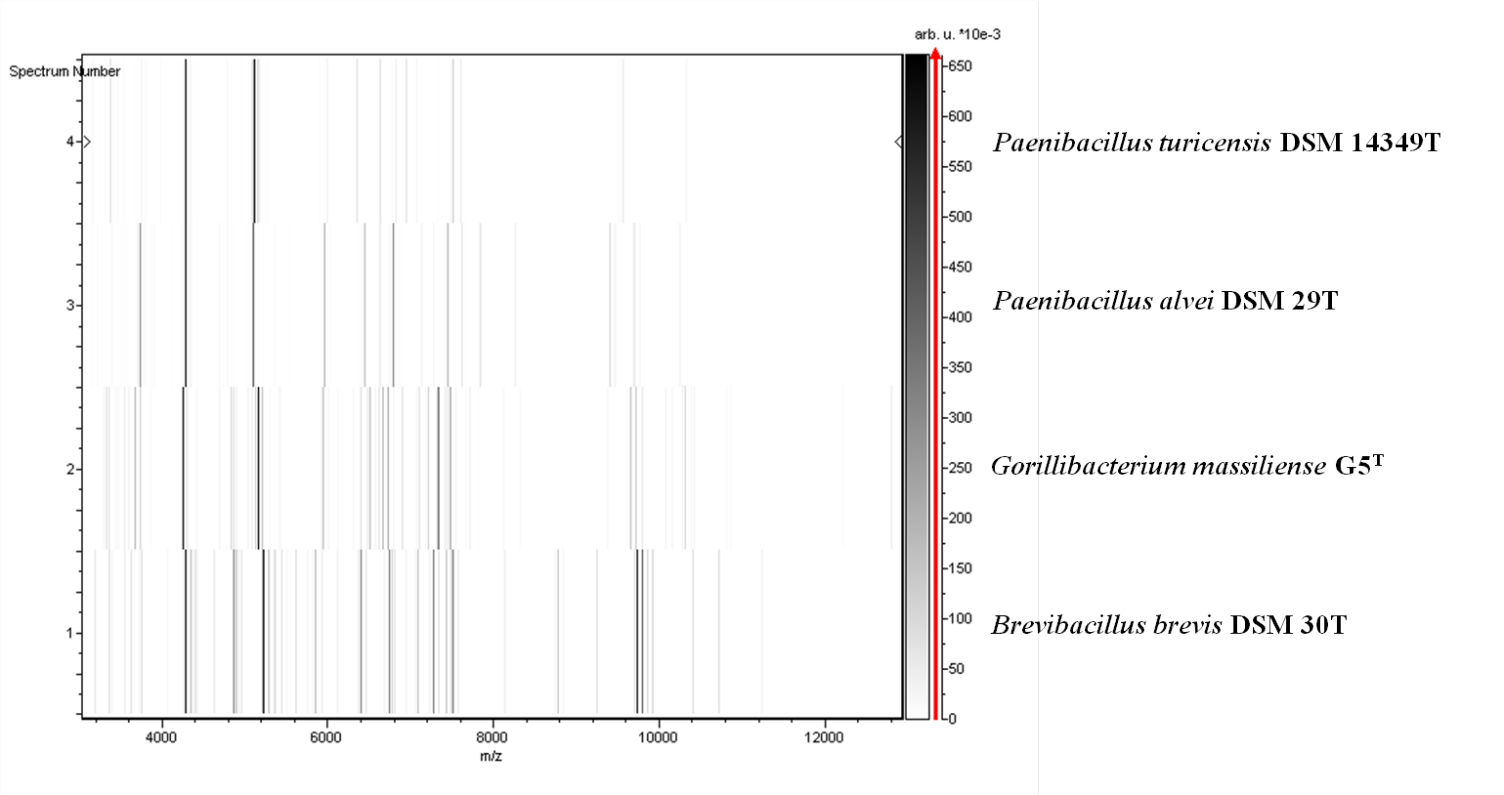
Gel view comparing *Gorillibacterium massilinensis* gen. nov., sp. nov strain G5^T^ spectra with other members of the *Paenibacillaceae* family. The Gel View displays the raw spectra of all loaded spectrum files arranged in a pseudo-gel like look. The x-axis records the m/z value. The left y-axis displays the running spectrum number originating from subsequent spectra loading. The peak intensity is expressed by a Gray scale scheme code. The color bar and the right y-axis indicate the relation between the color a peak is displayed with and the peak intensity in arbitrary units. Displayed species are indicated on the left.

Matrix-assisted laser-desorption/ionization time-of-flight (MALDI-TOF) MS protein analysis was carried out as previously described [15] using a Microflex spectrometer (Bruker Daltonics, Leipzig, Germany). Twelve distinct deposits were done for strain G5^T^ from 12 isolated colonies. The 12 G5^T^ spectra were imported into the MALDI BioTyper software (version 2.0, Bruker) and analyzed by standard pattern matching (with default parameter settings) against 6,252 bacterial spectra used as reference data, in the BioTyper database. A score enabled the presumptive identification of the isolated based on the following heuristicpecies: a score > 2 with a validated species enabled the identification at the species level, a score > 1.7 but < 2 enabled the identification at the genus level; and a score < 1.7 did not enable any identification. For strain G5^T^, a significant score was not obtained, suggesting it was not a member of any known species or genus. We incremented our database with the spectrum from strain G5^T^ (Figure 4). Spectrum differences with other of *Paenibacillaceae* family are shown in Figure 5.

## Genome sequencing information

### Genome project history

The organism was selected for sequencing on the basis of its phylogenetic position and 16S rRNA similarity to other members of the family *Paenibacillaceae*, and is part of a “culturomics” study of the gorilla flora aiming at isolating all bacterial species within gorilla feces. It was the 81^st^ genome of the *Paenibacillaceae* family and the first genome of *Gorillibacterium massiliense* gen. nov., sp. nov. A summary of the project information is shown in Table 3. The Genbank accession number is CBQR000000000 and consists of 176 large contigs. Table 3 shows the project information and its association with MIGS version 2.0 compliance [43].

**Table 3:** Project information

**MIGS ID**	**Property**	**Term**
MIGS-31	Finishing quality	High-quality draft
MIGS-28	Libraries used	454 paired-end 3- kb libraries
MIGS-29	Sequencing platform	454 GS FLX Titanium
MIGS-31.2	Sequencing coverage	25.71×
MIGS-30	Assemblers	Newbler version 2.5.3
MIGS-32	Gene calling method	Prodigal
	EMBL Date of Release	August 07, 2013
	EMBL ID	CBQR000000000
MIGS-13	Project relevance	Study of the gorilla gut microbiome

### Growth conditions and DNA isolation

*Gorillibacterium massiliense* gen. nov., sp. nov., strain G5^T^ (= CSUR P290 = DSM 27179) was grown aerobically on 5% sheep blood-enriched Columbia agar at 37°C. Four petri dishes were spread and resuspended in 3×500µl of TE buffer and stored at 80°C. Then, 500 µl of this suspension were thawed, centrifuged 3 minutes at 10,000 rpm and resuspended in 3×100 µL of G2 buffer (EZ1 DNA Tissue kit, Qiagen). A first mechanical lysis was performed by glass powder on the Fastprep-24 device (Sample Preparation system, MP Biomedicals, USA) using 2×20 seconds cycles. DNA was then treated with 2.5µg/µL lysozyme (30 minutes at 37°C) and extracted using the BioRobot EZ1 Advanced XL (Qiagen). The DNA was then concentrated and purified using the Qiamp kit (Qiagen). The yield and the concentration were measured by the Quant-it Picogreen kit (Invitrogen) on the Genios Tecan fluorometer at 50ng/µl.

### Genome sequencing and assembly

The paired-end library was prepared with 5 µg of bacterial DNA using DNA fragmentation on a Covaris S-Series (S2) instrument (Woburn, Massachusetts, USA) with an enrichment size at 4.5kb. DNA fragmentation was visualized with an Agilent 2100 BioAnalyzer on a DNA labchip 7500. The library was constructed according to the 454 GS FLX Titanium paired-end protocol (Roche). Circularization and nebulization were performed and generated a pattern with an optimum at 510 bp. After PCR amplification through 17 cycles followed by double size selection, the single stranded paired-end library was quantified using a BioAnalyzer 2100 on a RNA pico 6000 labchip at 68 pg/µL. The library concentration equivalence was calculated as 2.45E+08 molecules/µL. The library was stored at -20°C until further use.

The paired-end library was clonally amplified with 0.25 cpb and 0.5 cpb in 2 emPCR reactions with the GS Titanium SV emPCR Kit (Lib-L) v2 (Roche). The yield of the emPCR was respectively of 5 and 6% as expected of the yield ranging from 5 to 20% recommended by the Roche procedure.

Approximately 790,000 beads were loaded twice (i.e. two runs were performed using the same paired-end library) on a ¼ region of the GS Titanium PicoTiterPlate PTP Kit 70×75 and sequenced with the GS FLX Titanium Sequencing Kit XLR70 (Roche). The two runs were performed overnight and then analyzed on the cluster through the gsRunBrowser and Newbler assembler (Roche). A total of 387,157 passed filter wells were obtained and generated 142.7 Mb of sequences with a length average of 369 bp. The passed filter sequences were assembled using Newbler with 90% identity and 40-bp as overlap. The final assembly identified 12 scaffolds with 176 large contigs (>1.5kb), generating a genome size of 5.5 Mb which corresponds to a genome coverage of 25.71×.

### Genome annotation

Open Reading Frames (ORFs) were predicted using Prodigal [44] with default parameters but the predicted ORFs were excluded if they spanned a sequencing gap region. The predicted bacterial protein sequences were searched against the GenBank database [45] and the Clusters of Orthologous Groups (COG) databases using BLASTP. The tRNAScanSE tool [46] was used to find tRNA genes, whereas ribosomal RNAs were found by using RNAmmer [47] and BLASTn against the GenBank database. ORFans were identified if their BLASTP *E*-value was lower than 1e-03 for alignment length greater than 80 amino acids. If alignment lengths were smaller than 80 amino acids, we used an *E*-value of 1e-05.

To estimate the mean level of nucleotide sequence similarity at the genome level between *G. massiliense* and another 2 members of the family *Paenibacillaceae* and *Brevibacillus brevis*, we use the Average Genomic Identity of Orthologous gene Sequences (AGIOS), a custom application we developed. Briefly, the AGIOS software combines the Proteinortho software [48] for detecting orthologous proteins between genomes compared two by two, then retrieves the corresponding genes and determines the mean percentage of nucleotide sequence identity among orthologous ORFs using the Needleman-Wunsch global alignment algorithm.

## Genome properties

**Figure 6:**
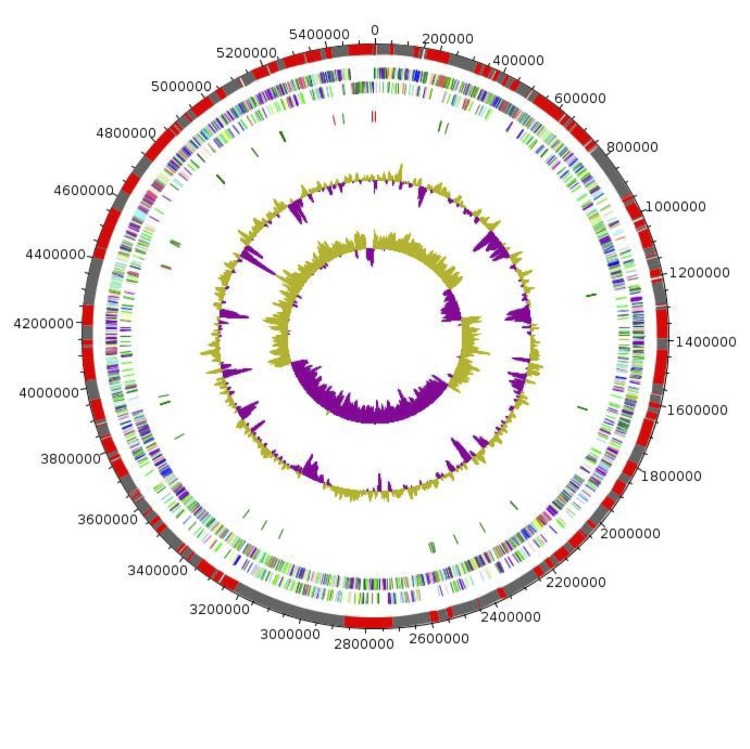
Graphical circular map of the chromosome. From outside to the center: Genes on the forward strand colored by COG categories (only genes assigned to COG), genes on the reverse strand colored by COG categories (only gene assigned to COG), RNA genes (tRNAs green, rRNAs red), G+C content and GC skew. Purple and olive indicating negative and positive values, respectively.

The genome is 5,546,433 bp long with a 50.39% G+C content (Figure 6 and Table 4). It is composed of 189 Contigs (176 large contigs, 12 scaffolds). Of the 5,221 predicted genes, 5,145 were protein-coding genes, and 76 were RNAs (1 gene is 16S rRNA, 1 gene is 23S rRNA, 5 genes are 5S rRNA, and 69 are tRNA genes). A total of 3,865 genes (75.12%) were assigned a putative function (by cogs or by NR blast). In addition, 272 genes were identified as ORFans (5.29%). The remaining genes were annotated as hypothetical proteins (680 genes => 13.22%). The distribution of genes into COGs functional categories is presented in Table 5. The properties and the statistics of the genome are summarized in Table 4 and 5.

**Table 4:** Nucleotide content and gene count levels of the chromosome

**Attribute**	Value	% of total^a^
Genome size (bp)	5,546,433	100
DNA G+C content (bp)	2,794,611	50.39
DNA coding region (bp)	4,888,209	88.13
Total genes	5,221	100
RNA genes	76	1.46
Protein-coding genes	5,145	98.54
Genes with function prediction	3,865	75.12
Genes assigned to COGs	3,881	75.43
Genes with peptide signals	709	13.78
Genes with transmembrane helices	1,267	24.63

^a^ The total is based on either the size of the genome in base pairs or the total number of protein-coding genes in the annotated genome

**Table 5:** Number of genes associated with the 25 general COG functional categories

**Code**	**Value**	**% age**^a^	**Description**
J	201	3.91	Translation, ribosomal structure and biogenesis
A	0	0	RNA processing and modification
K	483	9.39	Transcription
L	166	3.23	Replication, recombination and repair
B	1	0.02	Chromatin structure and dynamics
D	43	0.84	Cell cycle control, mitosis and meiosis
Y	0	0	Nuclear structure
V	119	2.31	Defense mechanisms
T	313	6.08	Signal transduction mechanisms
M	205	3.98	Cell wall/membrane biogenesis
N	73	1.42	Cell motility
Z	5	0.1	Cytoskeleton
W	0	0	Extracellular structures
U	50	0.97	Intracellular trafficking and secretion
O	121	2.35	Posttranslational modification, protein turnover, chaperones
C	180	3.5	Energy production and conversion
G	560	10.88	Carbohydrate transport and metabolism
E	355	6.9	Amino acid transport and metabolism
F	93	1.81	Nucleotide transport and metabolism
H	130	2.53	Coenzyme transport and metabolism
I	108	2.1	Lipid transport and metabolism
P	248	4.82	Inorganic ion transport and metabolism
Q	113	2.2	Secondary metabolites biosynthesis, transport and catabolism
R	648	12.59	General function prediction only
S	328	6.38	Function unknown
-	1,264	24.57	Not in COGs

^a^ The total is based on the total number of protein-coding genes in the annotated genome

## Genomic comparison of *G. massiliense* and other members of the family *Paenibacillaceae*

The genome of *G. massiliense* strain G5^T^ was compared to those of *P. elgii* strain B69, *P. alvei* strain DSM 29 and *B. brevis* strain NBRC 100599 (Table 6A and Table 6B). The draft genome of *G. massiliense* is smaller in size than those of *P. elgii*, *P. alvei* and *B. brevis* (5.54 vs 7.96, 6.83 and 6.3 Mb respectively). *G. massiliense* has a lower G+C content than *P. elgii* (50.39% vs 52.6%) but higher than those of *P. alvei* and *B. brevis* (50.39% vs 45.9% and 47.3% respectively). The protein content of *G. massiliense* is lower than those of *P. elgii*, *P. alvei* and *B. brevis* (5,146 vs 7,597, 6,823 and 5,946 respectively) (Table 6 and Table 6B). In addition, *G. massiliense* shares 2,122, 1,846 and 1,716 orthologous genes with *P. elgii*, *P. alvei* and *B. brevis*, respectively (Table 6). The nucleotide sequence identity of orthologous genes ranges from 66 to 67.6% among previously published genomes, and from 65.3 to 68.7% between *G. massiliense* and other studied genomes (Table 6A and Table 6B). Table 6 summarizes the number of orthologous genes and the average percentage of nucleotide sequence identity between the different genomes studied.

**Table 6A:** Genomic comparison of *G. massiliense* gen. nov., sp. nov., strain G5^T^ with four other members of the family *Paenibacillaceae*^†^

**Species**	**Strain**	**Genome accession number**	**Genome size (Mb)**	**G+C content**
*Gorillibacterium massiliense*	G5^T^	CBQR000000000	5.54	50.39
*Paenibacillus elgii*	B69	AFHW00000000	7.96	52.6
*Paenibacillus alvei*	DSM 29	AMBZ00000000	6.83	45.9
*Brevibacillus brevis*	NBRC 100599	AP008955	6.3	47.3

**^†^**Species and strain names, GenBank genome accession numbers, sizes and G+C contents

**Table 6B:** Genomic comparison of *G. massiliense* gen. nov., sp. nov., strain G5^T^ with four other members of the family *Paenibacillaceae*^†^

	*G. massiliense*	*P. elgii*	*P. alvei*	*B. brevis*
*G. massiliense*	**5,146**	68.7	66.7	65.3
*P. elgii*	2,122	**7,597**	67.6	66.4
*P. alvei*	1,846	2,336	**6,823**	66
*B. brevis*	1,716	2,278	1,936	**5,946**

**^†^**Numbers of orthologous protein shared between genomes (lower left triangle), average percentage similarity of nucleotides corresponding to orthologous proteins shared between genomes (upper right triangle). Bold numbers indicate numbers of proteins per genome.

## Conclusion

On the basis of phenotypic, phylogenetic and genomic analyses, we formally propose the creation of *Gorillibacterium massiliense gen. nov.,* sp. nov., that contains the strain G5^T^. This bacterium has been found in stool sample of wild gorilla collected in Cameroon.

### Description of *Gorillibacterium* gen. nov.

*Gorillibacterium* (go.ri.li.bac.te.ri’um. gor.il.i NL gen fem, the genus name of the great ape; bac.ter’i.um N.L. neut. n., bacterium a rod; gorillibacterium a rod-shaped bacterium isolated from a gorilla).

Gram-negative rod. Facultatively anaerobic. Mesophilic. Non-motile. Oxidase negative, catalase positive. Positive for urease, nitrate reduction, α- and β-galactosidase, arginine dihydrolase, arginine arylamidase, and β-glucosidase. Habitat: gorilla gut. Type species: *Gorillibacterium massiliense*.

### Description of *Gorillibacterium massiliense* gen. nov., sp. nov.

*Gorillibacterium massiliense* (ma.si.li.en′se. L. gen. neut. n. *massiliense*, of Massilia, the ancient Roman name for Marseille, France, where the type strain was isolated).

*G. massiliense* is Gram-negative rod. Facultatively anaerobic. Mesophilic. Optimal growth is achieved at 37°C. Non-sporulating and non-motile bacterium. Colonies are bright gray and 0.5-1 mm in diameter on blood-enriched Columbia agar. Cells are rod-shaped and have a mean diameter of 0.67 µm and a mean length of 1.75 µm.

Catalase positive, oxidase negative. Using the API 20NE system, positive reactions are observed for nitrate reduction and urease reaction, but indole production was negative. Using the API 50CH system (BioMerieux), a positive reaction was obtained for the fermentation of D-xylose, D-glucose, D-fructose, D-mannose, N-acetylglucosamine, aesculin, salicin, D-cellobiose, D-maltose, D-lactose, D-melibiose, D-saccharose, D-trehalose, inulin, D-melezitose, D-raffinose, glycogen, gentiobiose, D-turanose, Methyl- αD-glucopyranoside and starch. Negative reactions are observed for glycerol, ribose, D-galactose, L-rhamnose, L-sorbose, dulcitol, inositol, D-mannitol, D-sorbitol, methyl-αD-mannopyranoside, D-arabinose, amygdalin, arbitin, potassium gluconate, potassium 2-cetogluconate, potassium 5-cetogluconate, adonitol and D-tagatose. Using the API ZYM system, positive reactions were observed for the production of naphthol-AS-BI-phosphohydrolase, α-galactosidase, β- galactosidase, β- glucosidase, Arginine arylamidase and Arginine dihydrolase. The production of α- glucosidase, β- glucuronidase, esterase lipase, leucine arylamidase and cystine arylamidase, valine arylamidase, glycine arylamidase, phenylalanine arylamidase, lipase, alkaline phosphatase, acid phosphatase, N-acetyl-β-glucosaminidase and a-chymotrypsin are negative. Susceptible to ticarcillin, amoxicillin, tobramycin, imipenem, vancomycin and rifampin but resistant to ceftazidime, colistin and metronidazole.

The G+C content of the genome is 50.39%. The 16S rRNA and genome sequences are deposited in GenBank under accession numbers KC193239 and CBQR000000000, respectively. The type strain G5^T^ (= CSUR P290 = DSM 27179) was isolated from the fecal flora of a *Gorilla gorilla gorilla* from Cameroon.
